# Metoprolol prevents chronic obstructive sleep apnea-induced atrial fibrillation by inhibiting structural, sympathetic nervous and metabolic remodeling of the atria

**DOI:** 10.1038/s41598-017-14960-2

**Published:** 2017-11-02

**Authors:** Li Sun, Sen Yan, Xiaoyu Wang, Shiqi Zhao, Hui Li, Yike Wang, Shuang Lu, Xinwen Dong, Jing Zhao, Shengzhu Yu, Minghui Li, Yue Li

**Affiliations:** 1 Department of Cardiology, the First Affiliated Hospital, Harbin Medical University, Harbin, 150001 Heilongjiang Province China; 2 Key Laboratory of Cardiac Diseases and Heart Failure, Harbin Medical University, Harbin, 150001 Heilongjiang Province China; 3 Northeast Agricultural University, Harbin, 150030 Heilongjiang Province China; 4 Institute of Metabolic Disease, Heilongjiang Academy of Medical Science, Harbin, 150086 Heilongjiang Province China

## Abstract

Chronic obstructive sleep apnea (OSA) may promote the development of atrial fibrillation (AF) by inducing atrial electrical and structural remodeling as well as autonomic nerve hyperinnervation. Here, we investigated the roles of metoprolol in regulation of atrial remodeling induced by chronic OSA. A canine model of chronic OSA was established by stopping the ventilator and closing the airway for 4 h/day every other day for 12 weeks, while metoprolol (5 mg·kg-1·day-1) was continuously administered. Using that model, we observed that increases in sympathetic sprouting and atrial structural remodeling were sharply inhibited by metoprolol. Moreover, metoprolol dramatically inhibited the impairment of atrial energy metabolism by activating the Sirt1-AMPK pathway. *In vitro*, metoprolol significantly activated the Sirt1-AMPK pathway in intermittent hypoxic and isoproterenol-treated HL-1 cells, and the effect was abolished by the coadministration of EX-527, an inhibitor of Sirt1 activation. In summary, metoprolol protects against chronic OSA-induced atrial remodeling. Our results suggest a new and feasible treatment strategy for AF induced by OSA.

## Introduction

Obstructive sleep apnea (OSA), a form of sleep-disordered breathing, is caused by obstruction of the upper airway^1,2^. The severity of OSA is measured by the apnea-hypopnea index (AHI), the frequency of apneas and hypopneas per hour of sleep. An AHI in the range of 5 ≤ AHI < 15 represents mild OSA, while 15 ≤ AHI < 30 represents moderate OSA, and AHI ≥ 30 represents severe OSA^3^. Our previous study demonstrated that atrial fibrillation (AF) inducibility and duration were dramatically increased in OSA canines, and we further found that atrial remodeling, manifested by increased apoptosis, fibrosis, and autonomic sprouting, promoted the development of a substrate for AF^4^. Instantaneous blocking of parasympathetic but not sympathetic neurotransmission decreased AF inducibility and AF duration after acute apnea. However, the roles of sympathetic hyperinnervation in atrial remodeling induced by chronic OSA are still unknown.

Notably, OSA can induce tissue metabolic dysfunction due to intermittent hypoxia, which is mainly characterized by impaired glucose and fatty acid (FA) utilization and may contribute to an increased risk of cardiovascular disease^5–8^. This disordered glucose and FA metabolism may provide a substrate for the development and maintenance of AF^9,10^. Hypoxia inactivates the Sirt1-AMPK pathway, which helps regulate metabolic imbalances under oxygen-deficient conditions and helps cells utilize glucose and survive under harmful conditions^11^. Moreover, decreased Sirt1 and AMPK activity mediates pathological cardiac structural remodeling^12,13^.

Randomized, controlled trials indicate that continuous positive airway pressure (CPAP) can reduce the cardiovascular risk induced by OSA^14^. However, 46–83% of OSA patients are nonadherent to the treatment^7,15^. Hence, there is an urgent need to identify an efficacious and comfortable strategy to treat atrial remodeling caused by OSA.

We previously found that metoprolol was effective in preventing ketamine-induced malignant arrhythmia^16^. β-Blockers combined with other anti-arrhythmic drugs significantly reduced recurrences in patients with persistent AF^17^. Moreover, β-blockers improved myocardial glucose and FA utilization and decreased oxygen consumed in heart failure patients^18^. Despite the reported benefits, the use of β-adrenergic receptor antagonists in OSA patients may be limited by their influence on apnea-induced bradycardia. A very recent study showed that a β1-adrenergic receptor antagonist attenuated apnea-induced increases in heart rate but did not potentiate apnea-induced heart rate decreases in patients with hypertension and untreated OSA^19^.

To our knowledge, the effect of metoprolol on OSA-induced atrial remodeling has never been systematically studied. Therefore, it is reasonable to hypothesize that metoprolol may have favorable effects on atrial remodeling induced by OSA and reduce the occurrence of AF.

## Results

### Metoprolol down-regulated AF inducibility and mean AF duration in OSA canines

**Figure 1:**
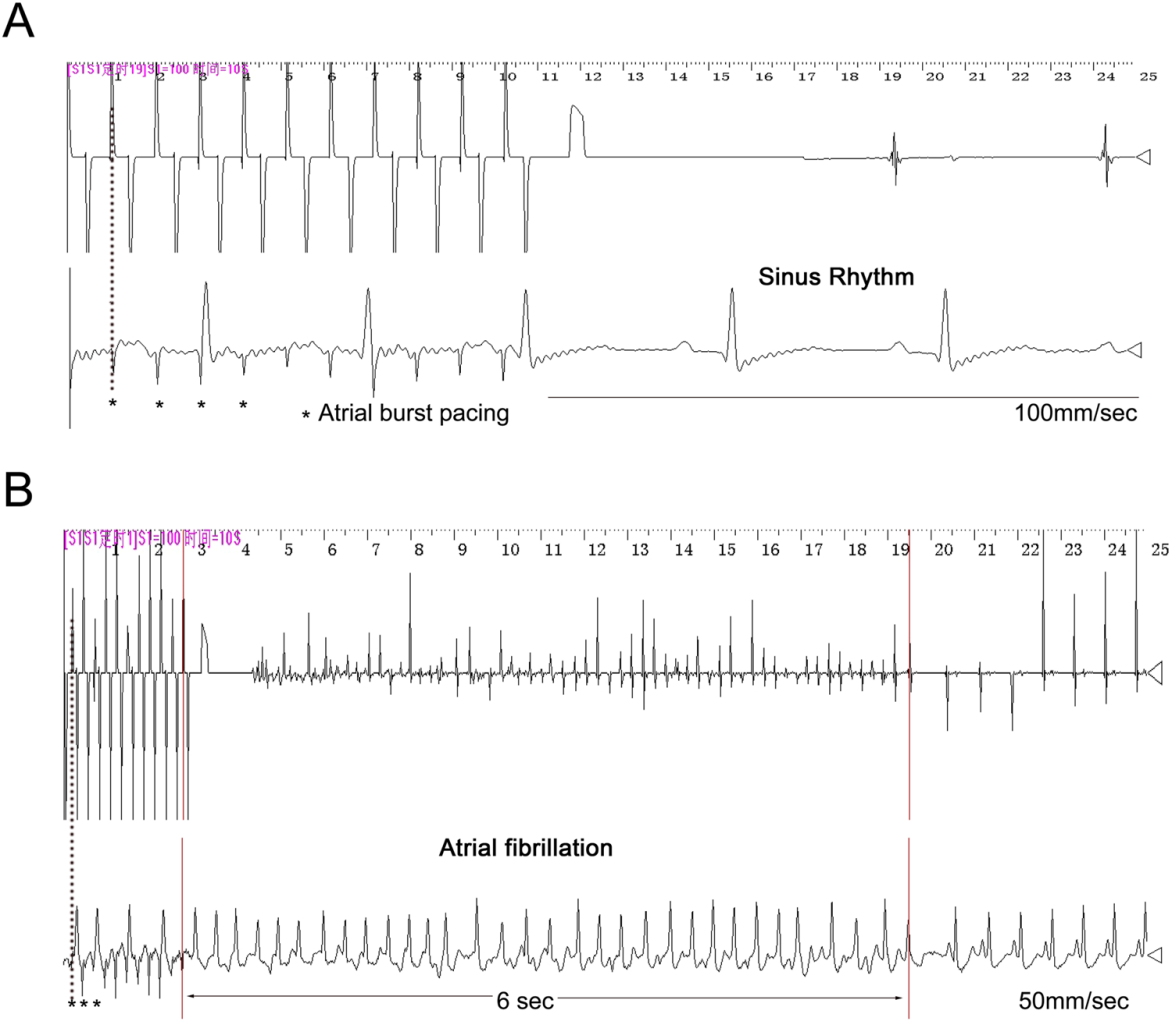
Examples of burst pacing-induced AF in sham-treated dogs (**A**) and OSA dogs (**B**).

**Figure 2:**
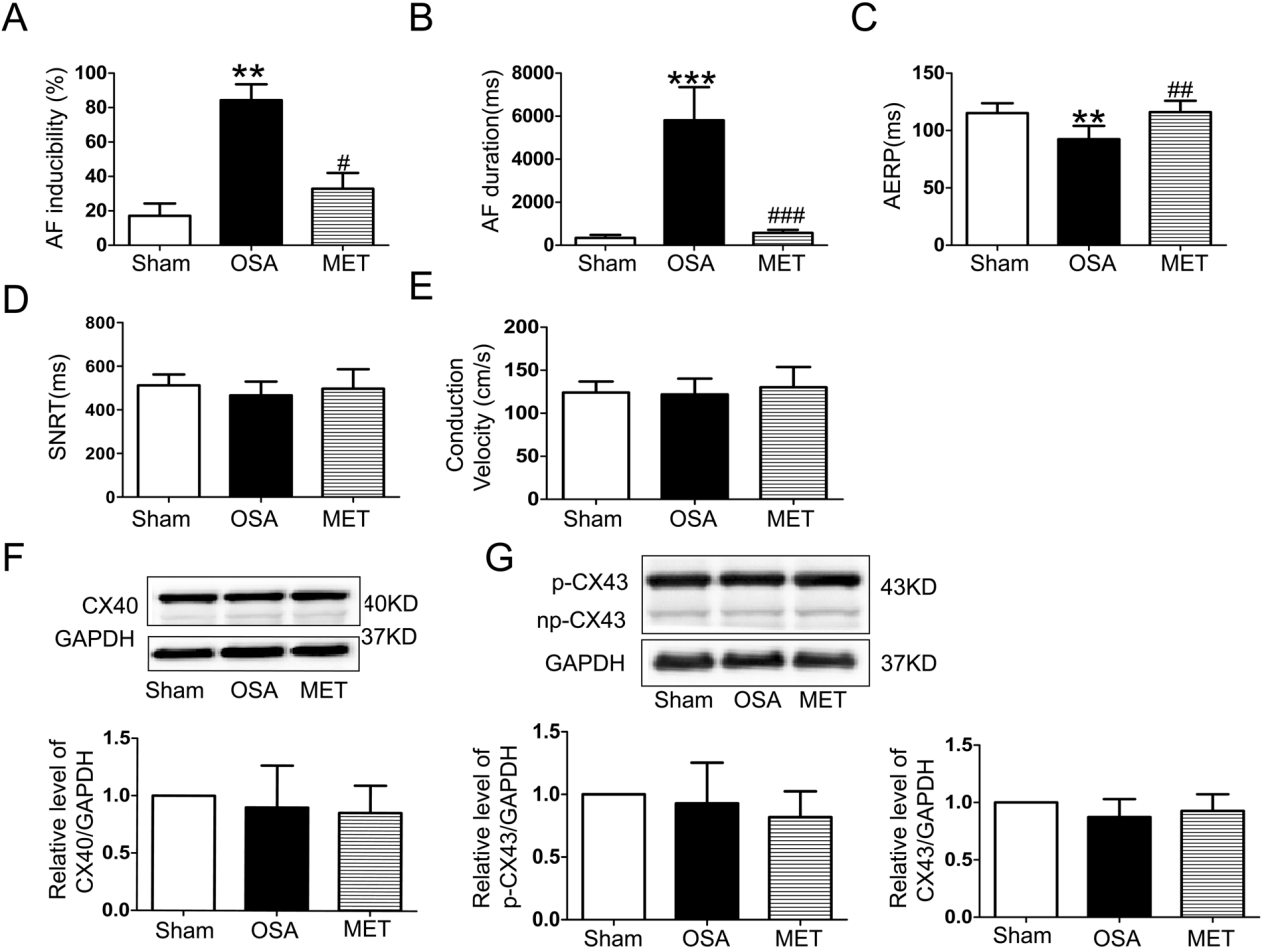
AF inducibility, AF duration, AERP, SNTR and intra-atrial conduction velocity in dogs from the Sham, OSA and MET groups. (**A**) AF inducibility in the Sham, OSA and MET groups. (**B**) Mean AF duration in the Sham, OSA and MET groups. (**C**) AERP in the Sham, OSA and MET groups. (**D**) Sinus node recovery time (SNRT) in the Sham, OSA and MET groups. (**E**) Intra-atrial conduction velocity in control and chronic OSA dogs. (**F**) Representative bands and statistical results of connexin (Cx) 40 in atrial tissue. (**G**) Representative bands and statistical results for Cx43 in the atrial tissue. *p < 0.05, **p < 0.01, ***p < 0.001 vs Sham group, ^#^ *p* < 0.05, ^##^ *p* < 0.01, ^###^ *p* < 0.001 vs OSA group, n = 7 per group.

Figure 1 shows recordings during typical burst pacing in OSA dogs and sham dogs. No arrhythmia was induced after burst cycles in sham dogs (Fig. 1A). In contrast, a long-lasting AF episode immediately followed burst cycles in OSA dogs (Fig. 1B). AF inducibility was significantly increased in chronic OSA dogs compared with that in dogs from the Sham group after tracheal blockage for 12 weeks. Similarly, mean AF duration was markedly prolonged in OSA dogs (Fig. 2). The atrial effective refractory period (AERP) of OSA dogs was lower than that of sham dogs, and this effect was prevented by metoprolol. Metoprolol treatment significantly decreased AF inducibility and mean AF duration (Fig. 2).

Additionally, no significant differences in intra-atrial conduction velocity or sinus node recovery time (SNRT) were observed among groups. The expression of total Cx40, total Cx43 and p-Cx43 did not significantly differ between the atria of chronic OSA dogs treated with and without metoprolol. Taken together, these results suggest that metoprolol inhibited AF caused by OSA.

### Metoprolol inhibited atrial sympathetic nerve remodeling caused by OSA

**Figure 3:**
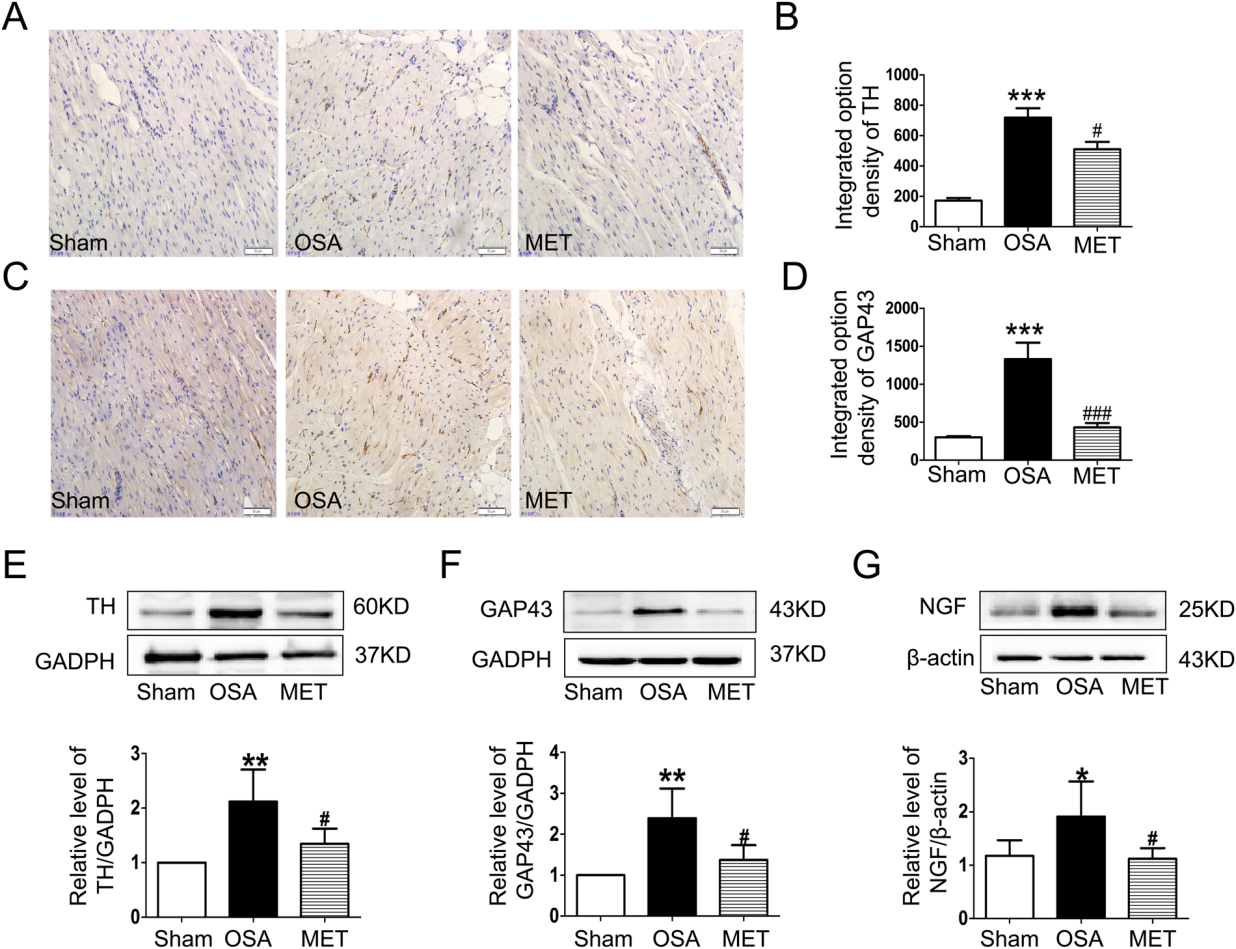
Sympathetic nerve sprouting and distribution of nerve fibers in the atria of OSA dogs and metoprolol-treated dogs. (**A**) Representative images of tyrosine hydroxylase (TH) expression in right atria. The magnification is 200 × (scale bar: 50 μm). (**B**) Statistical results for the expression of TH. (**C**) Representative images of GAP-43 expression in right atria. The magnification is 200 × (scale bar: 50 μm). (**D**) Statistical results for the expression of GAP43. (**E**–**G**) Representative bands and statistical results for the protein expression of TH, GAP43 and NGF in right atria. The expression levels of these proteins were normalized to the level of GAPDH or β-actin. **p < 0.01, ***p < 0.001 vs Sham group, ^#^ *p* < 0.05, ^##^ *p* < 0.01, ^###^ *p* < 0.001 vs OSA group, n = 6 per group.

We further examined sympathetic nerve remodeling in the atria of OSA dogs and found that chronic OSA induced extensive sympathetic nerve sprouting and changed the distribution of nerve fibers. The expression of GAP-43, a marker of newly formed nerve fibers, is induced during nerve sprouting. Nerve growth factor (NGF) is a neurotrophic factor that promotes the sprouting of sympathetic nerves. Tyrosine hydroxylase (TH) is an important marker expressed on sympathetic nerves. In right atria of OSA dogs, the densities of both TH- and GAP-43-positive fibers were higher than those in sham dogs, and this effect was inhibited by metoprolol (p < 0.05, Fig. 3A–D). Consistent with these results, the protein expression of TH, GAP43 and NGF was higher in OSA dogs than in sham dogs, and this elevation could be reduced by metoprolol (p < 0.05, Fig. 3E–G).

### Metoprolol inhibited atrial structural remodeling caused by OSA

After 12 weeks, the left atrial diameter (LAD), right atrial diameter (RAD), maximum left atrial volume (LAV_max_) and minimum left atrial volume (LAV_min_) of the OSA dogs increased compared with the 0-week baseline values, while the left atrial ejection fraction (LAEF) significantly decreased (p < 0.05, Table 1). No significant changes were observed in LAD, RAD, LAV_max,_ LAV_max_ or LAEF in sham dogs. Metoprolol treatment slightly increased LAD, RAD, LAV_max_ and LAV_max_ compared with their pre-apnea values, while there was no apparent decrease in LAEF (p > 0.05, Table 1).

**Table 1:** Transthoracic echocardiography examination of all the dogs at week 0 and week 12.

Group	CTRL (mean ± SD)		OSA (mean ± SD)		MET (mean ± SD)	
	week 0	week 12	week 0	week 12	week 0	week 12
LAD (mm)	24.9 ± 2.2	24.9 ± 2.3	25.3 ± 2.9	27.3 ± 2.7*	24.4 ± 2.8	24.4 ± 2.8
LAV_max_ (cm^3^)	9.2 ± 2.1	9.6 ± 1.6	11.3 ± 2.2	12.4 ± 2.8*	11.5 ± 3.1	11.8 ± 1.5
LAV_min_ (cm^3^)	4.5 ± 1.9	4.6 ± 2.3	5.1 ± 3.3	6.5 ± 4.1*	5.0 ± 1.7	5.3 ± 2.1
LAEF (%)	53.3 ± 3.5	54.2 ± 4.2	57 ± 4.7	50.6 ± 3.9*	57.7 ± 3.9	54.1 ± 8.2
RAD (mm)	20.7 ± 2.3	20.2 ± 2.2	23.5 ± 2.7	25.3 ± 3.1*	21.1 ± 2.5	23.3 ± 2.6
RAV_max_ (cm^3^)	3.7 ± 1.2	3.8 ± 1.6	3.9 ± 1.7	4.1 ± 1.6	3.8 ± 2.0	3.9 ± 2.6
RAV_min_ (cm^3^)	1.6 ± 0.3	1.5 ± 0.4	1.7 ± 0.6	1.8 ± 0.5	1.6 ± 0.4	1.7 ± 0.3

LAD, left atrial diameter; LAV_max_, maximum left atrium volume; LAV_min_, minimum left atrium volume; LAEF, left atrium ejection fraction; RAD, right atrial diameter; RAV_max_, maximum right atrium volume; RAV_min_, minimum right atrium volume. n = 4 per group, *p < 0.05, compared with week 0.

**Figure 4:**
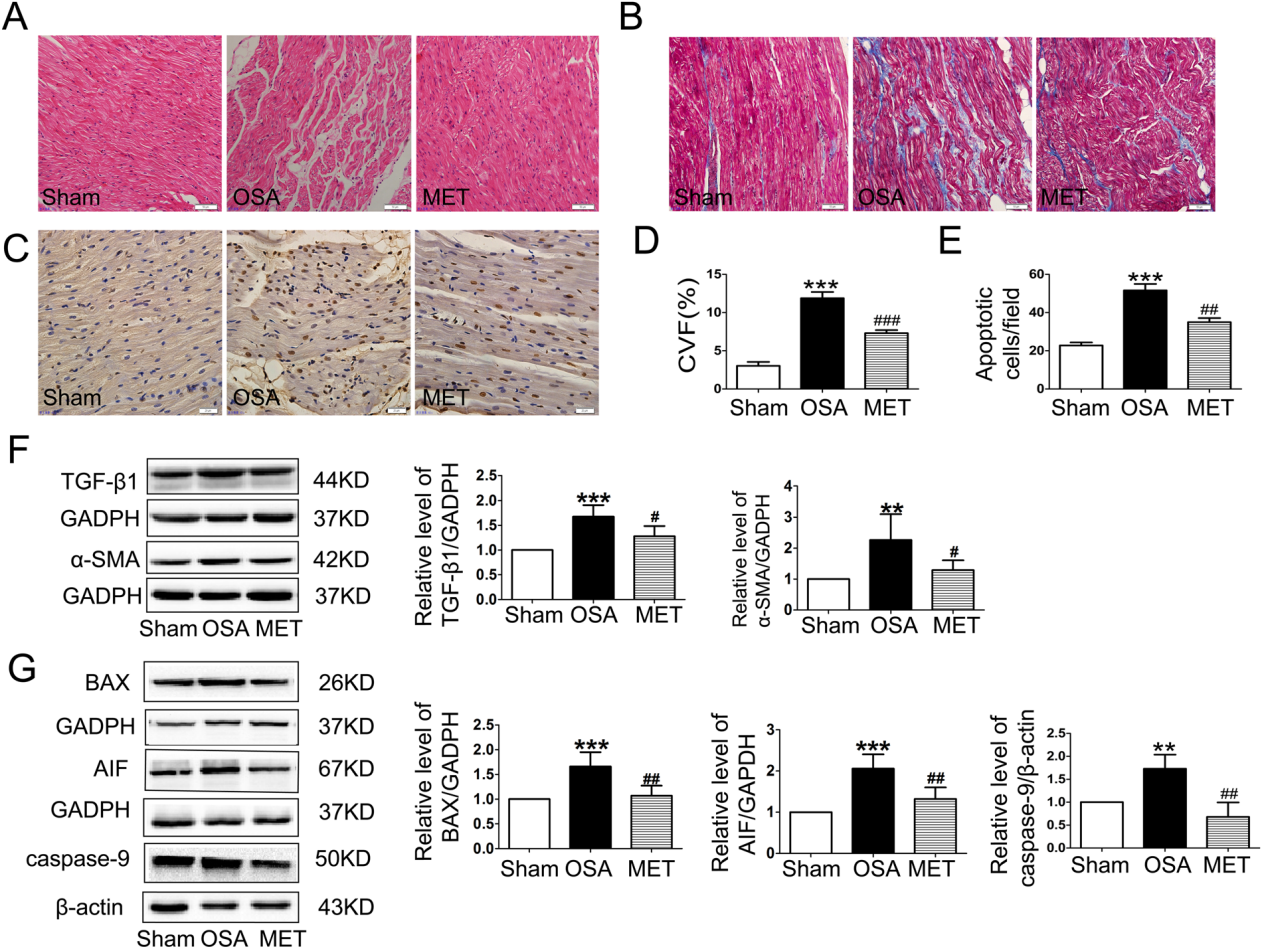
Structural changes after chronic OSA with or without metoprolol in canine right atrial myocytes. (**A**) Hematoxylin and eosin (H&E) staining, direct magnification: 200 × (scale bar: 50 μm). (**B**) Masson’s trichrome staining. The magnification is 200 × (scale bar: 50 μm). (**C**) TUNEL staining, magnification: 400 × (scale bar: 20 μm). (**D**) Collagen volume fraction (CVF) per field in the atria of dogs from each group. (**E**) Apoptotic cells per field in canine atria. (**F**) Representative bands and statistical results for the protein expression of TGF-β or α-SAM in canine atria. The expression levels of these proteins were normalized to the level of GAPDH. **p < 0.01, ***p < 0.001 vs Sham group, ^#^ *p* < 0.05, ^##^ *p* < 0.01, ^###^ *p* < 0.001 vs OSA group, n = 6 per group. (**G**) Representative bands showing protein expression of caspase-9, BAX and apoptosis-inducing factor (AIF). **p < 0.01, ***p < 0.001 vs Sham group, ^#^ *p* < 0.05, ^##^ *p* < 0.01, ^###^ *p* < 0.001 vs OSA group, n = 5 per group.

To observe the morphological changes in atrial myocytes in chronic OSA and metoprolol-treated dogs, we used hematoxylin and eosin (H&E) staining and transmission electron microscopy. As shown in Fig. 4A, the atrial myocytes of the sham dogs lined up tightly, surrounded by a small amount of connective tissue. Fibroblasts in the interstices were regular-sized and moderate in number. However, in OSA dogs, atrial myocytes were disordered, with exacerbated interstitial fibrosis. The changes in chronic OSA dogs were strongly inhibited by metoprolol. Similar results were found under transmission electron microscopy. Figure 7A shows that myofibrillae were irregular and ruptured in chronic OSA. The disappearance of sarcomeres was often limited to the vicinity of the nuclei. These changes were markedly ameliorated after the oral administration of metoprolol.

Furthermore, we used a terminal deoxynucleotidyl transferase dUTP nick-end labeling (TUNEL) assay to examine the effect of metoprolol on chronic OSA-induced atrial myocyte apoptosis. The percentage of labeled TUNEL-positive cells in the myocardium was significantly higher in chronic OSA dogs than in dogs in the Sham group (n = 5, Fig. 4C,E), and this change was prevented by metoprolol. To elucidate the molecular mechanism underlying the action of metoprolol, apoptosis-related proteins, including AIF, BAX and cleaved caspase-9, were assessed. We found that the expression of AIF, BAX, and cleaved caspase-9 was increased in chronic OSA, and the change was prevented by metoprolol treatment (Fig. 4G).

Masson staining was used to observe fibrosis in dogs from each group. Extensive interstitial fibrosis was observed in OSA dogs compared with that in sham dogs. The bundles of myofibers were packed less tightly in chronic OSA dogs than in sham dogs and were separated by thick layers of fibrous tissue; this effect was prevented by metoprolol (Fig. 4B,D). Additionally, fibrosis-related proteins, including TGF-β1 and α-SMA, were found to be increased in OSA dogs and were reduced by metoprolol (Fig. 4F).

### Metoprolol inhibited atrial metabolic remodeling caused by OSA

**Figure 5:**
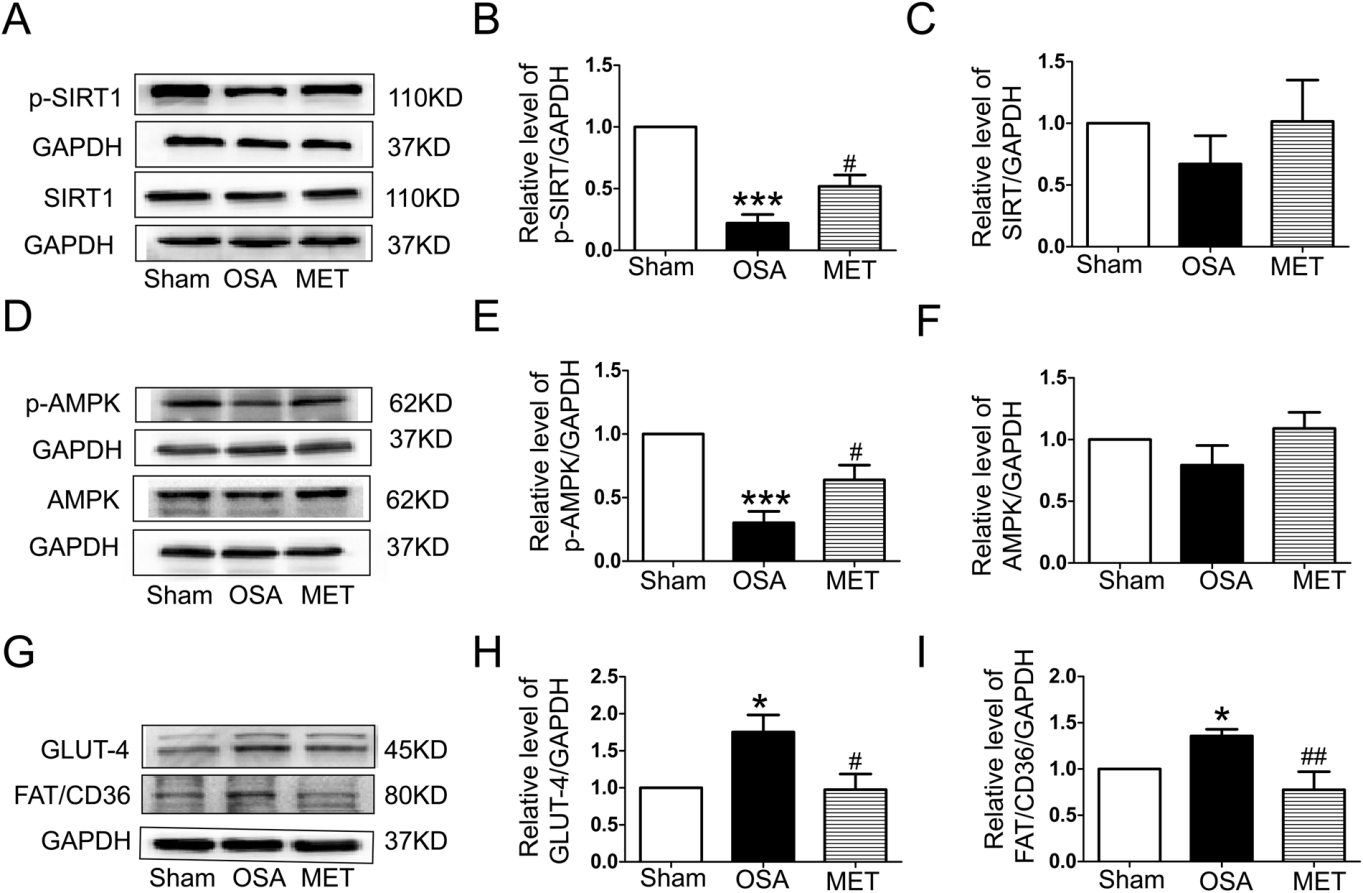
Expression of proteins involved in the Sirt1/AMPK pathway in atria of OSA dogs and metoprolol-treated dogs. (**A**) Representative bands showing the protein expression of p-Sirt1 and Sirt1 in right atria. (**B**,**C**) Statistical results for the expression of p-Sirt1 and Sirt1 in atrial tissue. (**D**) Representative bands showing the protein expression of p-AMPK and AMPK in right atria. (**E**,**F**) Statistical results for the expression of p-AMPK and AMPK in atrial tissue. (**G**) Representative bands for the protein expression of GLUT4 and FAT/CD36 in right atria. (**H**,**I**) Statistical results for the expression of GLUT4 and FAT/CD36 in atrial tissue. The expression levels of these proteins were normalized to the level of GAPDH. **p < 0.01, ***p < 0.001 vs Sham group, ^#^ *p* < 0.05, ^##^ *p* < 0.01, ^###^ *p* < 0.001 vs OSA group, n = 6 per group.

**Figure 6:**
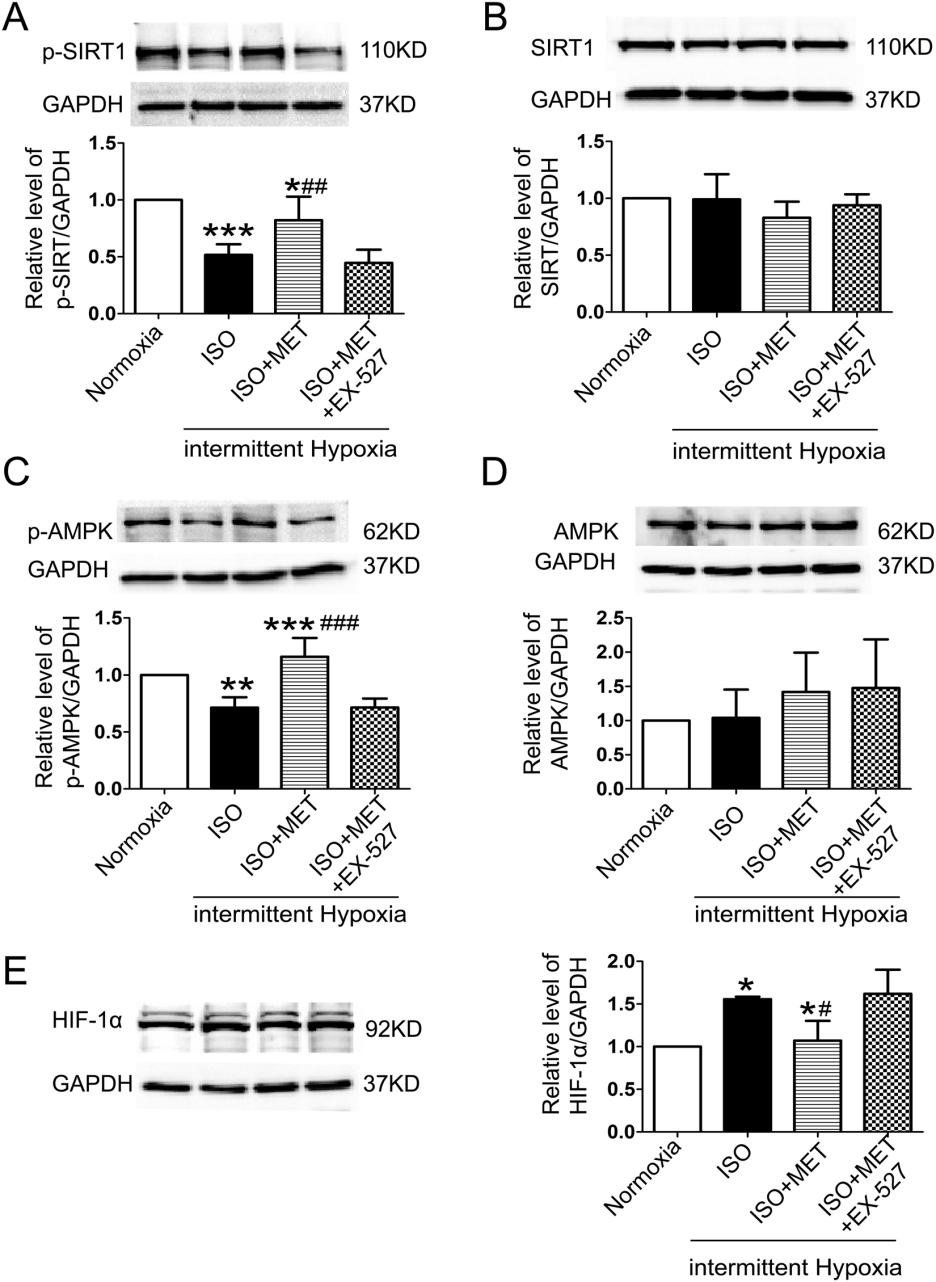
Expression of proteins involved in the Sirt1/AMPK pathway in hypoxic HL-1 cells. (**A**) Representative bands and statistical results for the protein expression of p-Sirt1 in HL-1 cells. (**B**) Representative bands and statistical results for the protein expression of Sirt1 in HL-1 cells. (**C**) Representative bands and statistical results for the protein expression of p-AMPK in HL-1 cells. (**D**) Representative bands and statistical results for the protein expression of AMPK in HL-1 cells. (**E**) Representative bands and statistical results for the protein expression of HIF-1α in HL-1 cells.The expression levels of these proteins were normalized to the level of GAPDH. *p < 0.05, **p < 0.01, ***p < 0.001 vs Normoxia group, ^#^ *p* < 0.05, ^##^ *p* < 0.01, ^###^ *p* < 0.001 vs the ISO + MET group, n = 4 each group.

To evaluate the effect and mechanism of action of chronic OSA on atrial energy metabolism, Sirt1 and AMPK were tested. We measured the levels of phospho-Thr172 AMPK α (an active catalytic subunit of the AMPK complex) in atria and found that the phosphorylation of Sirt1 and AMPK was decreased in OSA dogs, while metoprolol increased the activation of Sirt1 and AMPK (P < 0.05, Fig. 5A–F). Furthermore, the membrane expression of glucose transporter subtype 4 (GLUT4) and fatty acid translocase (FAT/CD36), two downstream targets of AMPK, was also measured. As shown in Fig. 5G, the total expression levels of GLUT4 and FAT/CD36 were up-regulated in the atria of OSA dogs compared with those in the atria of sham dogs. Metoprolol prevented the up-regulation of GLUT4 and FAT/CD36. Consequently, the impaired uptake of glucose and FAs was partially prevented by metoprolol via Sirt1 and AMPK activation. To confirm the potential role of metoprolol in the Sirt1-AMPK pathway, we used EX-527 to inhibit Sirt1 in intermittent hypoxic and isoproterenol (ISO)-treated HL-1 cells and examined the downstream AMPK activity. We found that the coapplication of EX-527 abolished the effect of metoprolol on AMPK activation (P < 0.05, Fig. 6).

### Metoprolol protects mitochondrial function against compromise by chronic OSA

**Figure 7:**
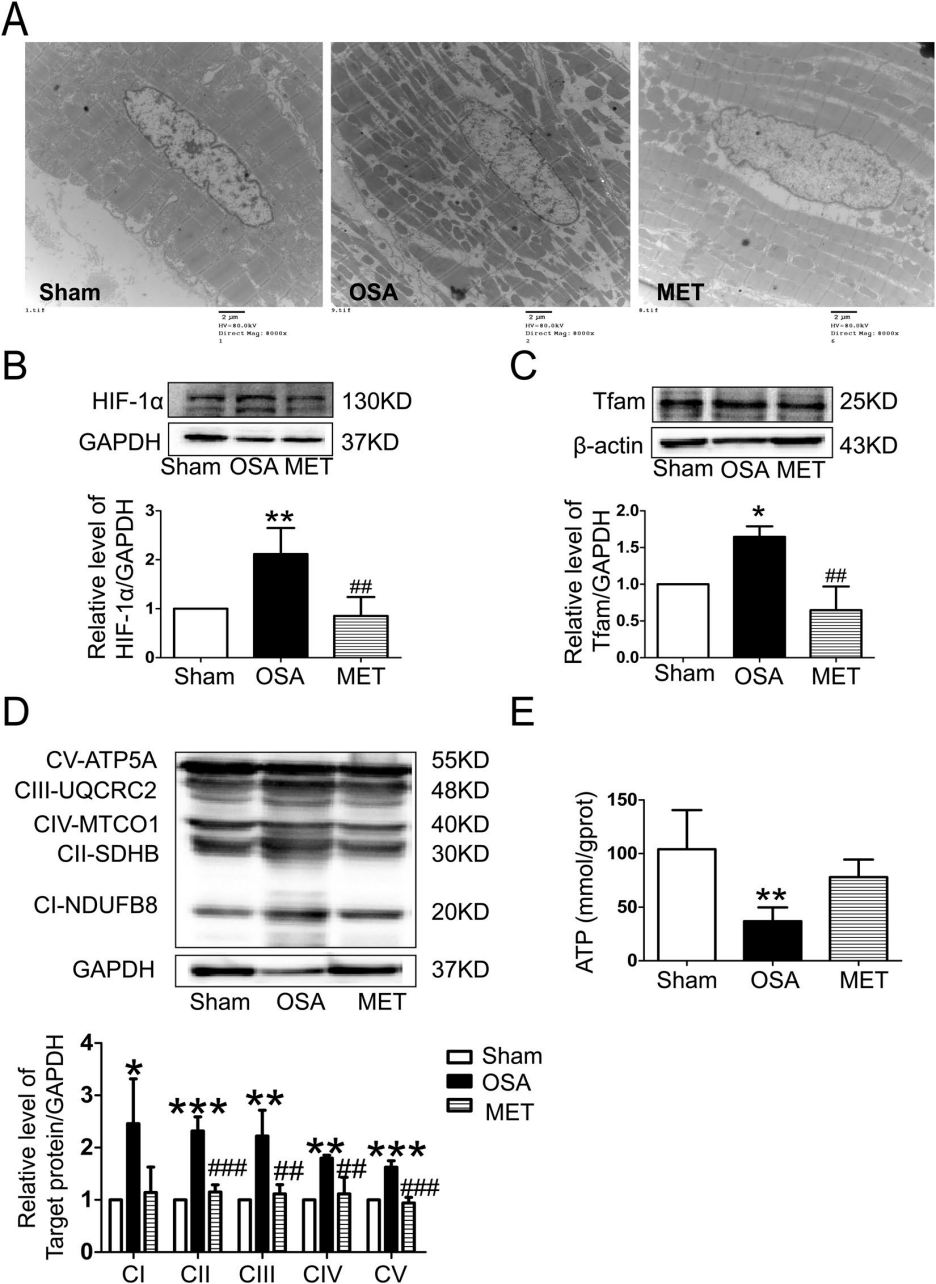
Protein levels of mitochondrial respiratory chain complexes and Tfam in atria of OSA dogs and metoprolol-treated dogs. (**A**) Transmission electron microscopy images in dogs from the Sham group, OSA group and MET group, direct magnification: 8000 × (scale bar: 2 μm). n = 6 each group. (**B**) Representative immunoblots of Tfam in atrial tissue and statistical results for Tfam. (**C**) Representative immunoblots of HIF-1α in atrial tissue and statistical results for Tfam. (**D**) Western blot results for NDUFB8, SDHB, UQCRC2, MTCO1, and ATP5A protein expression and the quantification of NDUFB8, SDHB, UQCRC2, MTCO1, and ATP5A protein levels. NDUFB8 indicates a subunit of nicotinamide-adenine dinucleotide dehydrogenase (complex I); SDHB, 30-kDa subunit of succinate dehydrogenase (complex II); UQCRC2, core protein 2 of ubiquinol-cytochrome *c* reductase complex (complex III); MTCO1, subunit I of cytochrome *c* oxidase (complex IV); ATP5A, a subunit of F _0_ F _1_-ATP synthase (complex V). The expression levels of these proteins were normalized to the level of GAPDH. **p < 0.01, ***p < 0.001 vs Sham group, ^#^ *p* < 0.05, ^##^ *p* < 0.01, ^###^ *p* < 0.001 vs OSA group, n = 5 per group. (**E**) The atrial tissue concentration of adenosine triphosphate (ATP) was measured using an ATP assay. The data are presented as the mean ± standard. **p < 0.01 vs Sham group, n = 4 per group.

Many swollen mitochondria were detected in the atria of OSA dogs (Fig. 7), suggesting severe mitochondrial injury induced by chronic OSA. Treatment with metoprolol preserved an optimally functioning mitochondrial pool by preventing the detrimental effects of chronic OSA injury. Our data show that hypoxia-inducible factor-1 alpha (HIF-1α), which decreases mitochondria oxidative phosphorylation in adaptation to hypoxia, was significantly increased in OSA canines. Metoprolol prevented hypoxia-induced increases in HIF-1α in atrial tissue and HL-1cells (Figs 6E and 7B). To complete our study, we examined mitochondrial biogenesis and the expression of mitochondrial respiratory chain complexes, which are critical to cardiac mitochondrial oxidative phosphorylation and consequent ATP production. The protein level of mitochondrial transcription factor A (Tfam) was significantly increased in the OSA group compared with that of the Sham group. The expression levels of a subunit of nicotinamide-adenine dinucleotide dehydrogenase (complex I; NDUFB8), a subunit of succinate dehydrogenase (complex II; SDHB), ubiquinol–cytochrome *c* reductase complex (complex III; UQCRC2), subunit I of cytochrome *c* oxidase (complex IV; MTCO1), and the α subunit of F0F1-ATP synthase (complex V; ATP5A) were higher in the OSA group than in the Sham group (P < 0.05). Metoprolol treatment decreased Tfam expression and mitochondrial respiratory chain protein synthesis (Fig. 7C,D). Together, these data suggest that OSA caused an atrial tissue energy deficiency, that mitochondrial biogenesis therefore increased to adapt to the hypoxic conditions, and that metoprolol prevented this energy deficiency and the resulting excessive mitochondrial synthesis.

## Discussion

In the current study, we found for the first time that metoprolol could effectively decrease the inducibility and duration of AF in a canine model of chronic OSA. Furthermore, we found that the following the mechanisms were involved: 1) inhibition of atrial sympathetic hyperinnervation with TH, GAP43 and NGF reduction; 2) inhibition of atrial myocyte apoptosis and fibrosis via the down-regulation of apoptosis- and fibrosis-related proteins, including cleaved caspase-9, AIF, BAX, α-SMA and TGF-β1 in chronic OSA canines; and 3) alleviation of atrial metabolic remodeling caused by OSA through the Sirt1-AMPK pathway.

The administration of metoprolol is disputed in OSA patients due to the concern that metoprolol may aggravate bradycardia induced by apnea^20^ or that a single-dose administration of non-selective β-blockers may cause airway narrowing in OSA patients^21^. However, researchers recently found that metoprolol, as a cardioselective β-blocker, seems to be safe for OSA patients^22^. Studies examining the effects of cardioselective β1-blockers found no consistently deleterious effect on lung function either acutely or with long-term use^23,24^. The impact of metoprolol on OSA-generated AF remains to be elucidated, and the available analyses have focused on the averaged sleep-time heart rate only^25^. Our data may explain in detail the mechanisms whereby metoprolol protects OSA patients from AF.

It is well known from previous clinical studies that OSA is closely related to AF^26,27^. Our studies have successfully established a canine model of chronic OSA and found that chronic OSA increased AF vulnerability. It is well known that atrial wavelength is dependent on the atrial refractory period and conduction velocity. Emanuele *et al*.^28^ observed that pretreatment with metoprolol helped prevent AF recurrence by contributing to AERP recovery after the electrical cardioversion of persistent AF in patients on amiodarone. Similarly, another double-blind, placebo-controlled study showed that metoprolol was effective in preventing relapse into AF after cardioversion in patients with persistent AF^29^. To date, it remains unknown whether metoprolol will prevent the onset of AF in OSA patients. In the present study, we found that AF inducibility was significantly decreased in the MET group and that the reduction in AERP induced by OSA was successfully prevented by metoprolol (Fig. 2). This evidence suggests that metoprolol decreased the vulnerability to AF, and thus, we further investigated the important underlying mechanisms.

A very recent clinical study found that apnea induced powerful and differentiated coactivation of the sympathetic and parasympathetic branches, which could lead to arrhythmias^22^. Additionally, chronic OSA increased autonomic nerve sprouting in the canine atrium^4^. The activation of the autonomic nervous system promotes the generation of AF substrates, and both sympathetic and parasympathetic activation differentially influence atrial electrophysiology^30^. Chronic rapid atrial pacing in dogs increased atrial sympathetic innervation and thus enhanced AF vulnerability^31^. Increased sympathetic nerve sprouting exacerbates electrophysiological heterogeneity and leads to a higher risk of ventricular arrhythmias and sudden cardiac death after myocardial infarction. Our previous research demonstrated that norepinephrine concentrations were significantly increased in the cardiomyocytes of OSA dogs^32^. Researchers found that metoprolol mediated an amelioration of sympathetic nerve sprouting in rabbits after myocardial infarction^33^. In the present study, we demonstrated that metoprolol significantly decreased atrial sympathetic nerve sprouting during chronic OSA (Fig. 3), which implies that sympathetic nerves may participate in the structural remodeling induced by OSA and thus decrease vulnerability to AF.

Kim *et al*.^34^ observed that the structural and functional remodeling of the left atrium was associated with the severity of OSA. Consistent with that study, the present study detected atrial enlargement and dysfunction in chronic OSA canines (Table 1). Atrial structural changes, including fibrosis and chamber enlargement, are associated with intra-atrial conduction velocity, which might play a critical role in the maintenance of AF. In the metoprolol-treated dogs, the atrial enlargement and dysfunction induced by OSA were prevented. To clarify the potential mechanism responsible for these changes, we further analyzed the histopathology and ultrastructural changes of the atrium. The deranged myofibers and swollen mitochondria in the cardiomyocytes of OSA dogs were ameliorated by metoprolol (Figs 4 and 7A). Furthermore, our previous study demonstrated that metoprolol could prevent the cardiotoxicity of ketamine, probably by reducing myocardial apoptosis. Apoptosis/fibrosis marker proteins, including cleaved caspase-9, AIF, BAX, α-SMA and TGF-β1, play important roles in the occurrence and maintenance of AF^16^. Therefore, we measured the expression of these proteins, and we were the first to demonstrate directly the protective effect of metoprolol against the complex atrial structure changes that occur in association with chronic OSA (Fig. 4).

Glycogen overload leads to atrial remodeling, conduction system disease and atrial arrhythmias. GLUT4 is the major glucose transporter in the myocardium; consequently, an increase in its expression level causes an increase in glucose uptake^35^. Increasing atrial glycogen is also associated with pacing-induced AF in dogs^36^. FA metabolism accounts for the majority of ATP production in the normal heart, and FAT/CD36 is responsible for 50–70% of FA uptake^37^. Increased membrane translocation of FAT/CD36 causes the supply of FAs to exceed the oxidative capacity of the cell; consequently, FAs are converted to lipids such as triglycerides and ceramides that have been described as causes of lipotoxicity, which may in turn cause cytotoxicity^38^. Sirt1 acts as a redox sensor to cope with metabolic imbalance under nutrient- or oxygen-deficient conditions^39^. Overall, Sirt1 helps cells utilize glucose and survive under harmful conditions. Sirt1 activates AMPK by deacetylating and activating the AMPK activator LKB1^40^. For the first time, we demonstrated the decreasing activity of Sirt1 and AMPK in the atria of chronic OSA dogs (Fig. 5). *In vitro*, the Sirt1-AMPK axis was down-regulated in intermittent hypoxic and ISO-treated HL-1 cells. Metoprolol reversed this reduction, and the effect of metoprolol was abolished by treatment with EX-527 (an inhibitor of Sirt1, Fig. 6). AMPK mediates glycogen and FA metabolism during AF. Interestingly, it was found that although the fraction of phosphorylated AMPK was increased in paroxysmal AF, it was decreased in chronic AF. Concerns have been raised regarding whether alterations to AMPK in energy metabolism play a critical part in the progression of AF. Clinical studies have shown that metoprolol promotes glucose oxidation in patients with dilated cardiomyopathy^41^. Moreover, we were the first to find that metoprolol could reverse the down-regulation of the Sirt1-AMPK pathway caused by chronic OSA and thus ameliorate the accumulation of glycogen and FAs (Figs 5 and 6).

We further investigated mitochondrial biogenesis, which is critical to the cellular energy supply. Researchers observed that mitochondrial DNA copy number is lower in whole blood DNA of OSA subjects than in that of normal controls, reflecting mitochondrial dysfunction in OSA patients. However, in our study, the expression of Tfam and mitochondrial respiratory chain proteins were increased in OSA dogs, suggesting that mitochondrial biogenesis was increased, which may be partly explained by adaptive responses. Metoprolol treatment decreased the expression of Tfam and mitochondrial respiratory chain proteins and prevented an energy deficiency and excessive mitochondrial synthesis (Fig. 7).

## Conclusions

**Figure 8:**
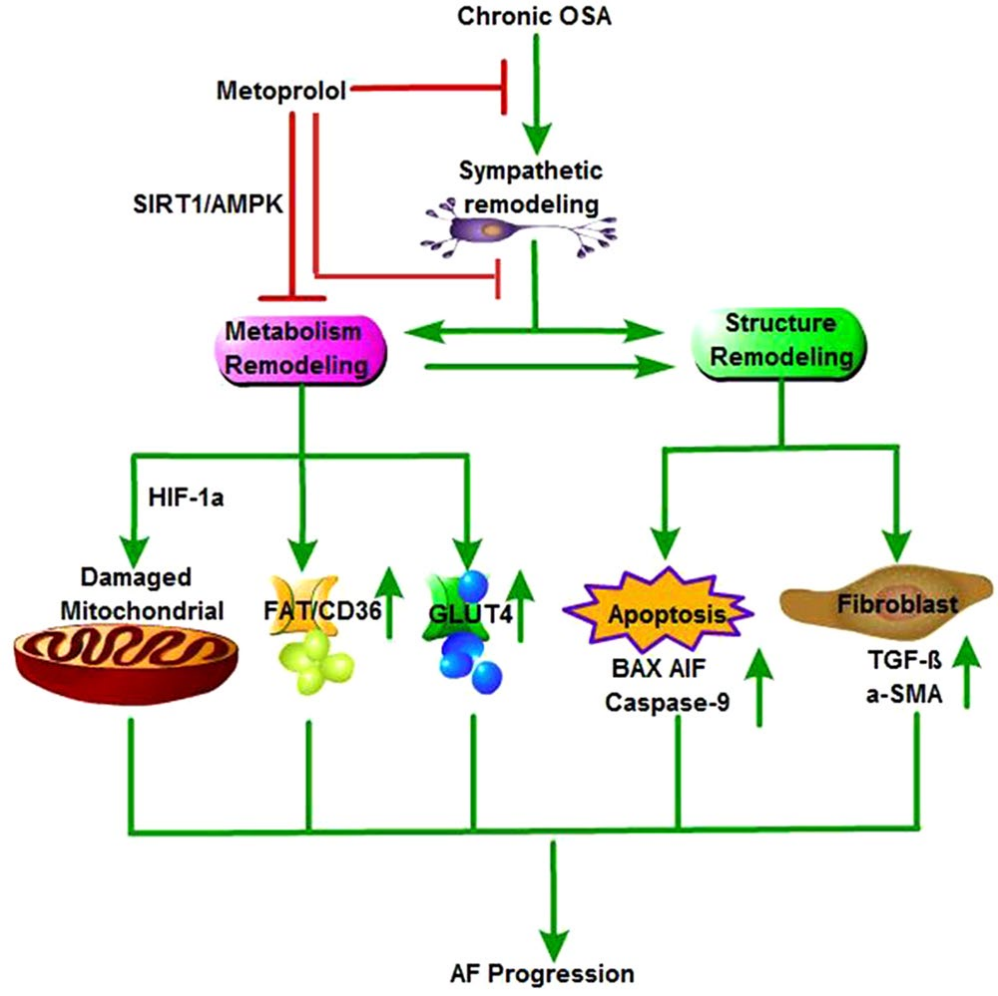
Schematic – activation of SIRT1/AMPK pathway by metoprolol is required to inhibit AF induced by chronic OSA. Chronic OSA causes structural, sympathetic nerve and metabolic remodeling of the atria. Metoprolol inhibits sympathetic hyperinnervation, acts as a positive modulator of Sirt1 activity, and initiates an adaptive response. (i) Sirt1/AMPK can act as a potent suppressor of HIF-1α and cause mitochondrial damage; (ii) decreased expression of FAT/CD36 and GLUT4 on the cell membrane was reversed, thus rescuing impaired uptake of glucose and FA; and (iii) metoprolol inhibits atrial apoptosis and fibrosis, perhaps partly through the Sirt1/AMPK pathway.

In summary, our study demonstrates that metoprolol significantly inhibits structural, sympathetic neural and metabolic remodeling in the atria of chronic OSA dogs (Fig. 8). Our findings highlight the potential utility of metoprolol in treating atrial remodeling, decreasing AF inducibility, and shortening the duration of AF. The above findings provide crucial support for the use of metoprolol to treat OSA patients.

## Methods

### Animals

Twenty-one healthy male mongrel dogs (15-20 kg, Experimental Animal Center of the First Affiliated Hospital of Harbin Medical University) were used in the study. The dogs were housed in the Experimental Animal Center of the First Affiliated Hospital of Harbin Medical University in a standard laboratory environment. The dogs were fed in individual cages in a temperature-controlled room at 23 °C under a 12:12 h light-dark cycle. All experimental procedures were approved by the ethics committees of Harbin Medical University and were compliant with the Guide for the Care and Use of Laboratory Animals published by the US National Institutes of Health (8th edition, 2011). After adaptive feeding for one week, the dogs were randomly divided into three groups: a Sham group (n = 7), an OSA (n = 7) group and an OSA- and metoprolol-treated (5 mg/kg/d) group (MET n = 7), metoprolol was administered starting from the beginning of the experiment. The Sham group underwent anesthesia and tracheal intubation only. Anesthesia was conducted according to the methods of a previous study^4^. Briefly, the dogs were anesthetized with ketamine (5.3 mg/kg, iv), diazepam (0.25 mg/kg, iv), and xylazine (1 mg/kg, iv). The adequacy of anesthesia was monitored based on the disappearance of the corneal reflex and jaw tone. All dogs were kept under anesthesia for 4 h during the chronic OSA periods and were drug-free for the remainder of the time.

### OAS stimulation

The tracheal tubes of anesthetized dogs were clamped to induce apnea at the end of the exhalation. The protocol conformed to that of our previous study. In short, the chronic OSA model was conducted for 12 weeks. In the first week, the duration of the trachea blockage was 1 min, and ventilation lasted 9 min. Thus, the apnea-hypopnea index (AHI) was set as 6. For the next three weeks, the duration of the trachea blockage was reduced by 1 min each week. Finally, the duration of trachea ventilation was 5 min with an AHI setting of 10 for the following 8 weeks. The entire procedure was performed for 4 h every other day^32^.

### Echocardiography

All dogs from each group underwent transthoracic echocardiography examination at week 0 and week 12 with a Philips 7500 apparatus. LAD, RAD, LAV_max_, LAV_min_, maximum right atrial volume (RAV_max_), minimum right atrial volume (RAV_min_) and left atrial ejection fraction (LAEF) were measured and recorded.

### Electrophysiological study

The electrophysiological measurements were conducted immediately after chronic airway obstruction for 12 weeks according to the methods of our previous study^42,43^. AERP was measured by performing 8 basic (S1) stimulations followed by a premature stimulus (S2). The interval between S1 and S2 was increased in 2-ms steps until capture no longer occurred. Continuous atrial pacing with 10 s and 100 ms intervals repeated 10 times was conducted to assess the inducibility and duration of AF. AF inductibility was defined as the relative ratio of successful induction frequency to total frequency of artial burst pacing in each group × 100%^43,44^. Intra-right atrium conduction velocity is expressed as the ratio of the distance between the upper and lower regions of the right atrium to the intra-atrial conduction time^45^.

### Histopathology and Masson trichrome staining

After the electrophysiological study, under general anesthesia the heart rapidly removed, and atrial tissues were prepared for histological and biochemical evaluation. Part of right atria tissue samples was fast frozenin liquid nitrogen and stored at −80 °C. Besides, part of right atria were collected in 4% paraformaldehyde and then embedded in paraffin. The tissues were cut into 5 μm sections and stained with H&E and Masson trichrome for histological and collagen analysis. Histopathological changes were observed via light microscopy. The fibrotic areas were calculated, and the collagen volume fraction (CVF) was quantified as the % = collagen area/total area × 100^16^. Additionally, part of each right atrium was fixed in 2.5% glutaraldehyde and sliced according to the standard procedure. Sections were then rinsed in buffer, stained with uranyl acetate, dehydrated in ethanol, embedded in epoxy resin, and photographed with a transmission electron microscope (JEOL, Tokyo, Japan).

### Immunohistochemical assessment

Immunohistochemistry was performed on paraffin-embedded atrial sections. Slices were incubated with anti-TH (Abcam) and anti- GAP43, Abcam) overnight at 4 °C. The slides were treated with peroxidase-conjugated goat anti-rabbit IgG (Zhongshan, China) at 37 °C for 20 min.

### TUNEL assay

Formalin-fixed, paraffin-embedded sections were stained for apoptotic cells using a TUNEL assay according to the manufacturer’s instructions (Roche, Indianapolis, IN, USA). The nuclei of TUNEL-positive cells were stained brown, while TUNEL negative cells showed blue nuclei under an Olympus BX-60 microscope (Olympus, Tokyo, Japan). The number of apoptotic atrial cells was counted, and the apoptotic rate was expressed as a percent of total atrial cells.

### Cell culture

HL-1 cardiac cells were purchased from the Institute of Biochemistry and Cell Biology, Chinese Academy of Science, China. HL-1 cells were cultured in Claycomb medium (JRH Biosciences, USA) with 10% fetal bovine serum (Gibco, Invitrogen) and 1% penicillin-amoxicillin.

### Hypoxic treatment

HL-1 cells treated with isoproterenol (2 μM), metoprolol (10 μM) and EX-527 (4 µM) were placed in a sealed hypoxia chamber (Billups-Rothenberg) equilibrated with certified gas containing 1% O_2_, 5% CO_2_, and 94% N_2_ for 10 minutes and then switched to gas containing 21% O_2_, 5% CO_2_,74% N_2_ for 10 minutes. The total intermittent time was 12 hours. The intermittent hypoxia unit was set according to the previous study^46^. The control cells were exposed to 12 hours of normoxia.

### Determination of adenine triphosphate content

The atrial tissue concentration of adenosine triphosphate (ATP) was measured using an ATP assay kit according to the manufacturer’s standard procedure (Nanjing Jiancheng Bioengineering Institute, Nanjing, China). To correct for any variability in the amount of tissue, the measurements were normalized to the overall protein amount as measured by a bicinchoninic acid (BCA) assay.

### Western blotting

Right atrial proteins were harvested using RIPA buffer containing 1% protease inhibitor. The proteins were separated by size using 10% SDS-polyacrylamide gel electrophoresis and then transferred onto polyvinylidene difluoride membranes. The membranes were blocked with 10% nonfat milk for 1 h and then incubated with primary antibodies against Cx40, Cx43, caspase-9, BAX, AIF, TGF-β1, NGF, GAP43, TH, Sirt1/p-Sirt1, AMPK/p-AMPK, GLUT4, FAT/CD36, HIF-1α, Tfam or a MitoProfile Total OXPHOS Rodent WB Antibody Cocktail overnight at 4 °C. On the following day, the membranes were incubated with the secondary antibody for 1 h. An electrochemiluminescence kit was used to develop the chemiluminescent signals, which were detected with a ChemiDoc XRS gel documentation system (Bio-Rad, Hercules, CA). The protein bands were analyzed with Image Lab software.

### Statistical comparison

All data are presented as the mean ± SD. Multiple group comparisons were performed via one-way ANOVA followed by Tukey’s tests. The statistical analysis was conducted with GraphPad Prism 5.0 software, and p < 0.05 was considered statistically significant.

## Electronic supplementary material

Supplementary Information
