# Supplementary material for: Metoprolol prevents chronic obstructive sleep apnea-induced atrial fibrillation by inhibiting structural, sympathetic nervous and metabolic remodeling of the atria
Source: Sci Rep. 2017 Nov 2;7:14941. doi: 10.1038/s41598-017-14960-2 (PMC5668297; doi:10.1038/s41598-017-14960-2)
Supplement: Supplementary file 1 — Supplementary Information [file 41598_2017_14960_MOESM1_ESM.doc]

**Metoprolol** **prevents chronic obstructive sleep apnea-induced atrial fibrillation by** **i****nhibiting** **structural, sympathetic** **nervous and metabolic remodeling of the atria**

Li Sun^1^*, Sen Yan^1^*, Xiaoyu Wang^1^*, Shiqi Zhao^1^, Hui Li^1^, Yike Wang^1^, Shuang Lu^1^, Xinwen Dong^1^, Jing Zhao^2^, Shengzhu Yu^3^, Minghui Li^1^, Yue Li ^1, 2, 4 †^

Running title: Metoprolol prevents AF induced by chronic OSA

^1^Department of Cardiology, the First Affiliated Hospital, Harbin Medical University, Harbin 150001, Heilongjiang Province, China

^2^Key Laboratory of Cardiac Diseases and Heart Failure, Harbin Medical University, Harbin, 150001, Heilongjiang Province, China

^3^Northeast Agricultural University, Harbin, 150030, Heilongjiang Province, China

^4^Institute of Metabolic Disease, Heilongjiang Academy of Medical Science, Harbin, 150086, Heilongjiang Province, China

*These authors contribute equally to this paper.

* Corresponding author at: the First Affiliated Hospital, Harbin Medical University, Youzheng Street 23#, Nangang District, Harbin 150001, Heilongjiang Province, China.

Tel: +86 451 85555673; Fax: +86 451 53675733

E-mail address: ly99ly@vip.163.com (Y. Li)

**Supplemental Experimental Procedures:**

**Continual hypoxic treatment**

HL-1 cells treated with isoproterenol (2 μM), metoprolol (10 μM) and EX-527 (4 µM) were placed in a sealed hypoxia chamber (Billups-Rothenberg) equilibrated with certified gas containing 1% O_2_, 5% CO_2_, and 94% N_2_ for 12 hours. The control cells were exposed to 12 hours of normoxia.

**Supplemental Data:**


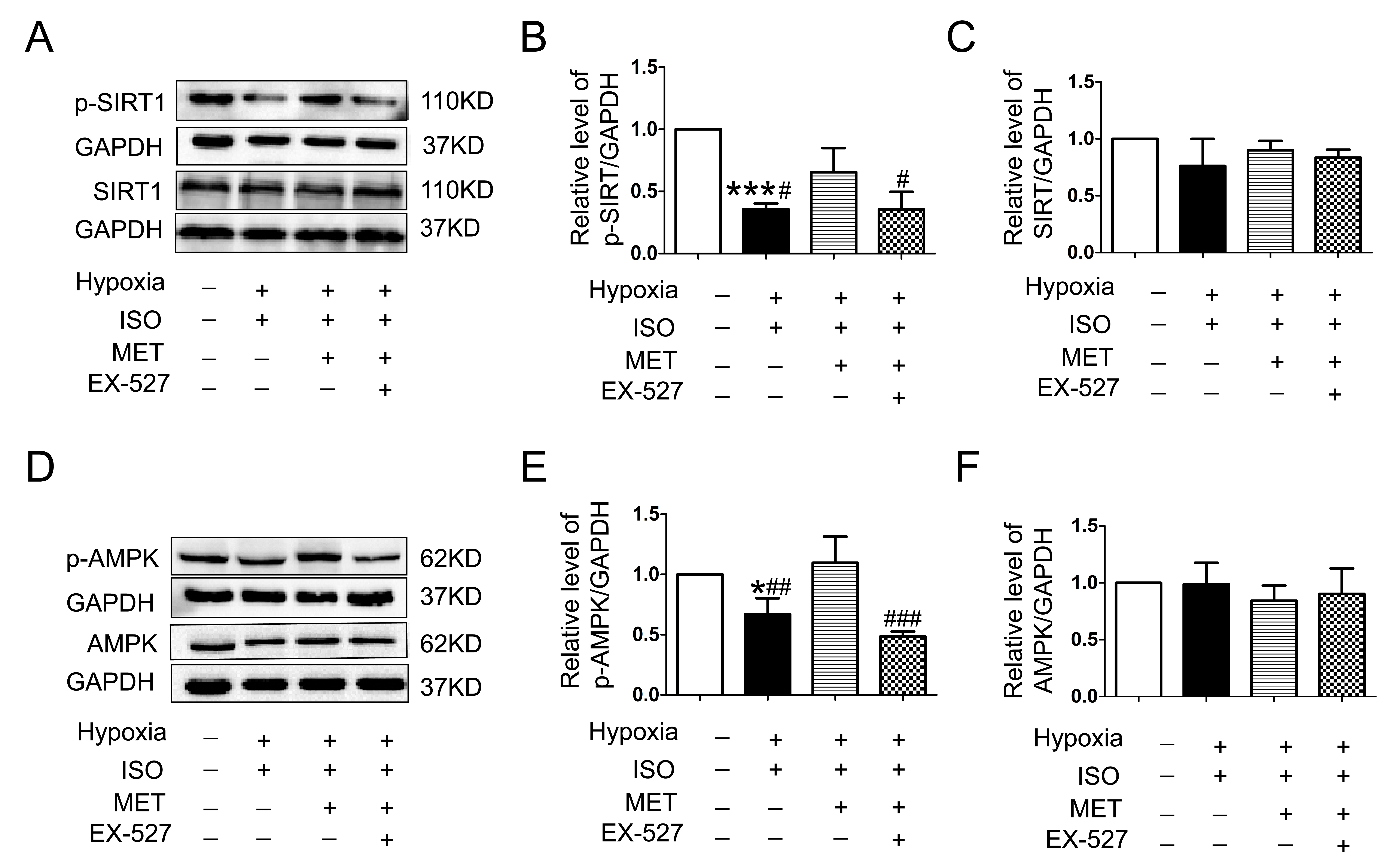


**Supplementary Fig S1 Expression of proteins involved in the Sirt1/AMPK pathway in** **continual hypoxic HL-1 cells.**

A) Representative bands showing the protein expression of p-Sirt1 and Sirt1 in HL-1 cells. B and C) Statistical results for the expression of p-Sirt1 and Sirt1 in HL-1 cells. D) Representative bands showing the protein expression of p-AMPK and AMPK in HL-1 cells. E and F) Statistical results for the expression of p-AMPK and AMPK in HL-1 cells. The expression levels of these proteins were normalized to the level of GAPDH or β-actin. **p*<0.05, ***p*<0.01, ****p*<0.001 vs Normoxia group, #*p*<0.05, ##*p*<0.01, ###*p*<0.001 vs the ISO+MET group, n=4 each group.


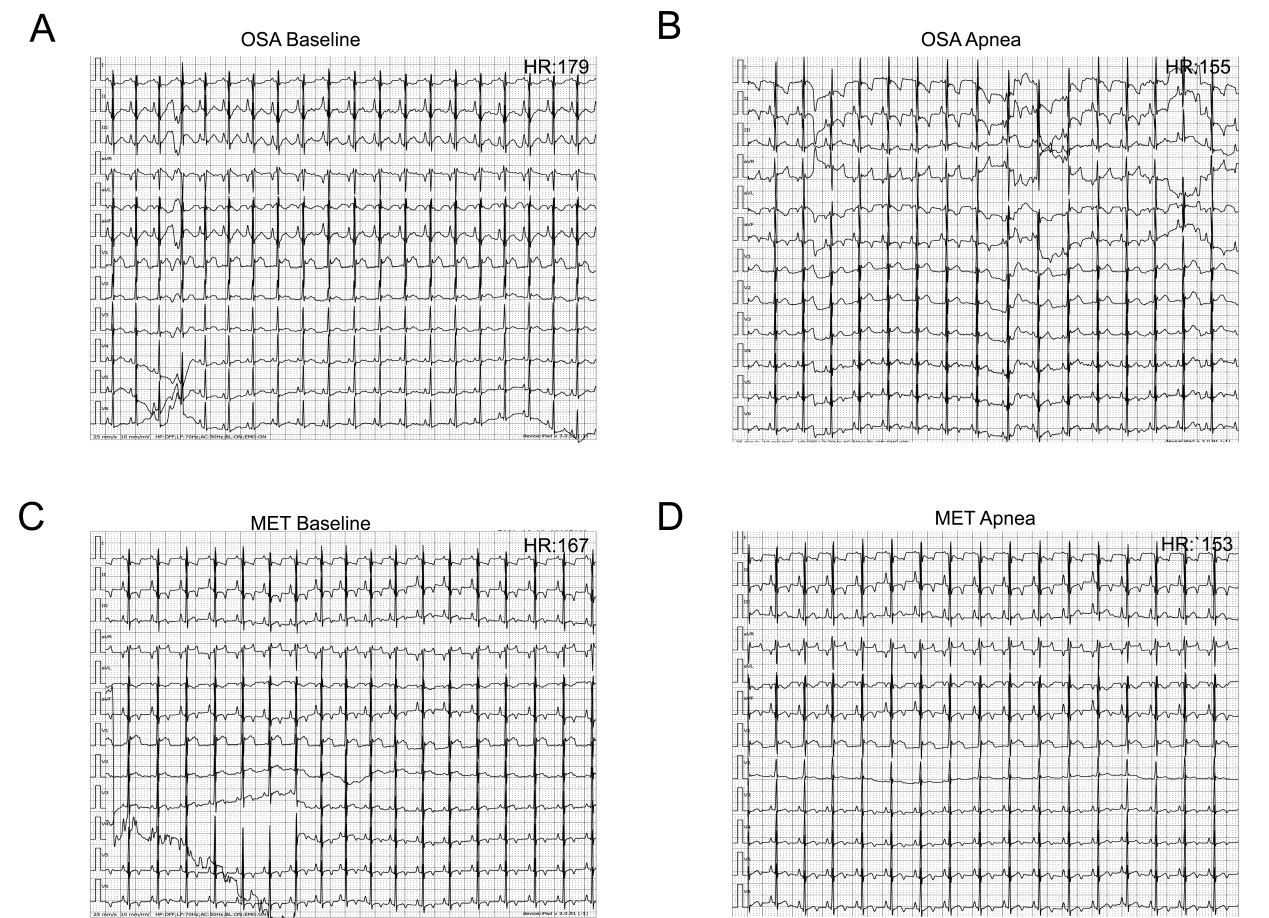


**Supplementary Fig S2 Repr****esentative** **electrocardiograms of dogs from OSA and MET group at week 12.**

1. Baseline electrocardiograms of dogs from OSA group. B)

Electrocardiograms of dogs from OSA group after apnea. C) Baseline electrocardiograms of dogs from MET group. D) Electrocardiograms of dogs from MET group after apnea.

**Table S1: Arteria** **blood gases and** **electrolytes of dogs from OSA and MET group** **at week 12**

| Group | OSA  Baseline | OSA  Apnea | MET  Baseline | MET  Apnea |
| --- | --- | --- | --- | --- |
| PH | 7.40±0.02 | 7.30±0.02** | 7.40±0.02 | 7.325±0.03** |
| PCO_2_ (mmHg) | 37.85±3.66 | 50.58±3.86* | 38.35±3.57 | 55.18±3.54** |
| PO_2_ (mmHg) | 90.5±1.29 | 43.2±3.60*** | 88.75±2.62 | 43±3.16*** |
| HCO^3-^(mmol/L) | 21.5±1.29 | 25.5±0.42** | 22.68±2.07 | 27.05±1.47* |
| SaO_2_ (%) | 96.36±0.3 | 65.92±10.0** | 95.92±0.17 | 64.58±7.72** |
| Na^+^ (mmol/L) | 143±3.91 | 144.25±3.09 | 142.5±3.87 | 145±3.56 |
| K^+^ (mmol/L) | 3.97±0.13 | 3.98±0.08 | 3.93±0.17 | 3.92±0.18 |
| Ca^2+^ (mmol/L) | 1.37±0.09 | 1.38±0.06 | 1.35±0.07 | 1.4±0.08 |
| BE (mmol/L) | -0.88±1.15 | -0.98±1.17 | -1.02±1.09 | -0.65±1.02 |

SaO_2_ oxygen saturation, BE base excess.*p<0.05, **p<0.01, ***p<0.001 vs. baseline, n=6.
